# Comparative Evaluation of Enalapril and Losartan in Pharmacological Correction of Experimental Osteoporosis and Fractures of Its Background

**DOI:** 10.1155/2013/325693

**Published:** 2013-01-16

**Authors:** D. S. R. Rajkumar, A. V. Faitelson, O. S. Gudyrev, G. M. Dubrovin, M. V. Pokrovski, A. V. Ivanov

**Affiliations:** ^1^Department of Traumatology and Orthopedics and Battle Field Surgery, Kursk State Medical University, Karl Marx Street, Kursk 305 004, Russia; ^2^Department of Clinical Pharmacology, Kursk State Medical University, Karl Marx Street, Kursk 305 004, Russia; ^3^Department of Pharmacology, Kursk State Medical University, Karl Marx Street, Kursk 305 004, Russia; ^4^Department of Histology, Cytology and Embryology, Kursk State Medical University, Karl Marx Street, Kursk 305 004, Russia

## Abstract

In the experiment on the white Wistar female rats (222 animals), the osteoprotective effect of enalapril and losartan was studied on experimental models of osteoporosis and osteoporotic fractures. It was revealed that in rats after ovariectomy, the endothelial dysfunction of microcirculation vessels of osteal tissue develops, resulting in occurrence of osteoporosis and delay of consolidation of experimental fractures. Enalapril and losartan prevented the reduction of microcirculation in bone, which was reflected in slowing the thinning of bone trabeculae and in preventing the occurrence of these microfractures, as well as increasing quality of experimental fractures healing.

## 1. Introduction

The blood flow plays a significant role in the process of bone remodeling and reparative regeneration of bone tissue [1, 2]. Microvessels of the bones have just the endothelium; they do not have the muscle and connective tissue layers. Consequently, it is the endothelium that mediates the entire humoral regulation of exchange between osteoblasts, osteoclasts, and the blood [3, 4]. At the same time in the available literature, we found no information that anyone has used the vascular endothelium of bone as a target for pharmacological action in osteoporotic changes.

In our opinion, one of the reasons for the deterioration of regional blood supply of bone tissue is the endothelial dysfunction, which causes negative effects on the microcirculation that could lead to a disturbance in the processes of bone formation and osteoreparation, thereby causing osteoporosis. The increase in the frequency of osteoporosis and its complications shows that there is currently no reliable methods of drug treatment and prevention of this disease. Modern pathogenetic therapy of osteoporosis has neglected drugs with favorable effects on blood flow to bone. This indicates the actuality of research actions about osteoprotective drugs with proven positive effects on endothelium.

## 2. Objective

To estimate the osteoprotective property of angiotensin-converting enzyme (ACE) inhibitor enalapril and angiotensin receptor blocker (ARBs) losartan on experimental models of osteoporosis and on the osteoporotic fractures.

## 3. Materials and Methods

The experiments were conducted for 222 white female Wistar rats weighing 200–300 g. All manipulations in the experiment were performed under general anesthesia (intraperitoneal injection of chloral hydrate solution at a dose of 300 mg/kg). To study, the animals are divided into 8 groups: (i) control (intact animals) (*n* = 42); (ii) after ovariectomy (*n* = 30); (iii) after ovariectomy daily intragastric administration of enalapril 0.5 mg/kg for 8 weeks (*n* = 35); (iv) after ovariectomy daily intragastric administration of losartan 6 mg/kg for 8 weeks (*n* = 35); (v) intact animals after osteotomy of the proximal metaphysis of femur (*n* = 20); (vi) animals in which ovariectomy was performed eight weeks after the model fracture of the proximal metaphysis of femoral was performed (*n* = 20); (vii) the animals with ovariectomy and intragastric administration of enalapril 0.5 mg/kg once daily, eight weeks after the model fracture of the proximal metaphysis of femur was performed (*n* = 20); (viii) the animals with ovariectomy and intragastric administration of losartan 6 mg/kg once daily, eight weeks after the model fracture of the proximal metaphysis of femur was performed (*n* = 20) (Tables 1 and 2).

Systemic osteoporosis develops by a bilateral ovariectomy [5]. To confirm this development until the last and to evaluate the effectiveness of study drug, eight weeks (57 days) after ovariectomy, bone histomorphometry was performed. Material was stained with hematoxylin-eosin (H-E). Slides from the histological preparations were subjected to light microscopy using a microscope Leica CME (zoom ×100, objective lens ×10, eyepiece ×10), and bone trabeculae were photographed with a digital camera Olympus SP-350 resolution 3264∗2448 pixel by comparing the camera lens and the eyepiece of the microscope. During bone histomorphometry used the program Image J, version 1.39, pre-calibration is done as follows: photographed a “scale” of 1 mm on a transparent substrate using the same equipment that is used for taking photographs of bone trabeculae, then measured the “scale” in pixels by program Image J, the length for 1425 pixels is 1 mm. Later measured the width of bone trabeculae, and expressed it in micrometers. In the first four groups 8 weeks after ovariectomy, the level of the microcirculation was measured in the proximal metaphysis of femur. For this, in the intertrochanteric area of the femur, a hole was drilled with a depth of 2.5–3 mm and 1 mm in diameter, into which the needle of sensor probe was introduced. To obtain data on the state of microcirculation in bone, equipment manufactured by Biopac systems were used: MP100 polygraph unit with laser Doppler flowmetry LDF100C and invasive needle probe TSD144. After defining the intra-osseous microcirculation level, without changing the probe position, were carried out on the samples the endothelium dependent vasodilation (EDVD) in response to a single intravenous injection of a solution of acetylcholine dose of 40 mg/kg [6] and endothelial nondependent vasodilation (ENVD) in response to a single intravenous injection of sodium nitroprusside solution at a dose of 30 mg/kg [7].

To study the osteoprotective effect, we selected drugs with a pronounced endothelium protective effect: enalapril 0.5 mg/kg and losartan 6 mg/kg [8–10]. Drugs were administered intragastrically once daily as a suspension in 1% starch paste. Animals which have not been treated received the same pattern of 1% starch paste.

In the next four groups of animals eight weeks after ovariectomy, transverse osteotomy of proximal metaphysis of the femur was performed. After modeling the fracture, intramedullary fixation was conducted by K-wire 1 mm in diameter. The results of the consolidation of fractures were analyzed visually and radiographically after four weeks; preliminarily, the parameters of microcirculation in the callus were checked in the manner specified earlier.

Statistical analysis was performed by means of Microsoft Excel from the data. “Descriptive statistics” was used to determine the average value indicators (*M*) and error of the mean (*m*). “Two-sample *t*-test with different variances” was used to compare the respective rates in different groups of animals and determine the differences between them. Statistically, significant differences in values of bilateral were considered *P* < 0.05. The relationship between different parameters within one group was assessed using the Pearson correlation coefficient (*r*), as well as through the construction of charts connecting the lines of regression. Given the impossibility of using the criterion *x* ^2^ (the expected value of contingency table 2 × 2 was less than five), to assess the reliability of the differences between the shares, double-sided version of Fisher's exact test was used.

## 4. Results and Discussion

**Figure 1:**
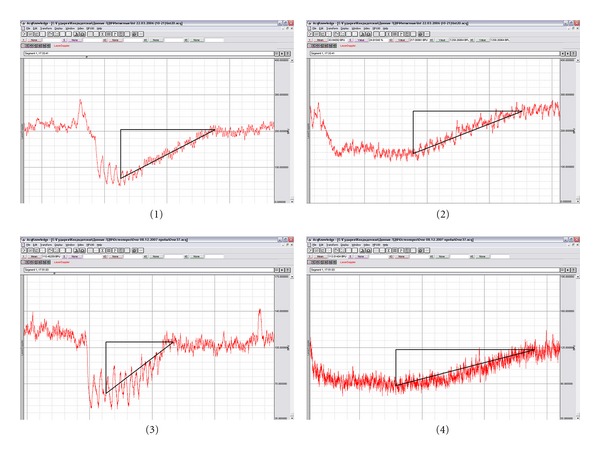
Examples of dynamics of the level of microcirculation in bone during functional vascular tests. (1) EDVD response in intact rats; (2) ENVD reaction in intact rats; (3) EDVD reaction in rats after bilateral ovariectomy; (4) ENVD reaction in rats after bilateral ovariectomy.

**Figure 2:**
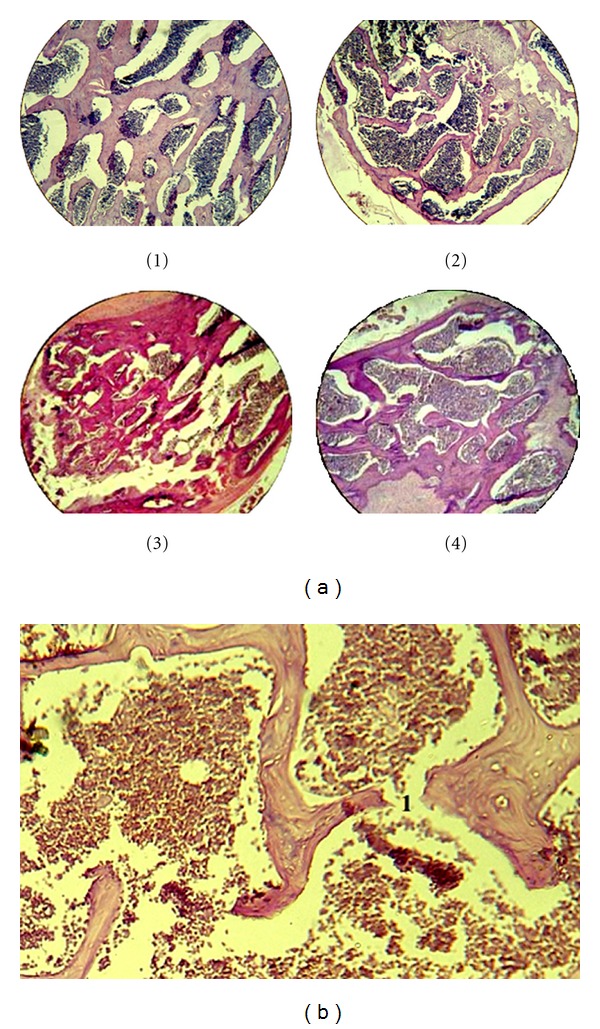
(a) The morphological picture of proximal metaphysis of femoral bone (strained with hematoxylin and eosin, SW-100). (1) In intact rats; (2) in the rat after ovariectomy; (3) in the rat after ovariectomy treated with enalapril; (4) in the rat after ovariectomy treated with losartan. (b) The morphological picture of proximal metaphysis of femoral bone in rats with generalized osteoporosis (strained with hematoxylin and eosin, SW-400). Microfracture of bone trabeculae.

Results of laser Doppler flowmetry (LDF) has allowed to state significantly lower level of the microcirculation in the bone tissue of rats eight weeks after ovariectomy (*n* = 30), 61.5 ± 3.7 perfusion units (PU) when compared with intact animals (*n* = 42), 100.5 ± 4.4 PU. It was found that a group of rats treated with enalapril 0.5 mg/kg (*n* = 35), index of microcirculation was 93.3 ± 4.4 perfusion units PU and the animals with losartan 6 mg/kg (*n* = 35), 100.0 ± 2.3 perfusion units PU. These figures suggest that these drugs are effective in preventing decline in regional blood flow in the femoral bone after ovariectomy. To confirm the role of endothelial dysfunction in development of disturbance in regional microcirculation, coefficient endothelial dysfunction (CED) was calculated based on data from LDF. In response, the systemic administration of acetylcholine and sodium nitroprusside shows decreased microcirculation with subsequent normalization of blood flow parameters (Figure 1). CED is defined as the ratio of area of the triangle over recovery curve of microcirculation in response to the introduction of sodium nitroprusside to the area the triangle over the recovery curve of microcirculation in response to the administration of acetylcholine. In the group of intact animals received CED = 1.3 ± 0.2, in the group of rats with osteoporosis CED = 2.4 ± 0.2. These results indicate the formation of changes; it is an evidence of endothelial dysfunction in microvessels of bone after ovariectomy. CED in rats treated with the studied drugs reduced up to 1.6 ± 0.1 for the enalapril and 1.5 ± 0.2 for losartan. Osteoporotic changes in the bones of the skeleton were confirmed histologically in all rats eight weeks after ovariectomy and were expressed by the thinning of bone trabeculae and increasing intertrabecular space (Figure 2(a)), also observed in histological preparations microfractures of bone trabeculae. The occurrence of microfractures was judged by the germination of connective tissue in the zone of fractured trabeculae (Figure 2(b)).

**Figure 3:**
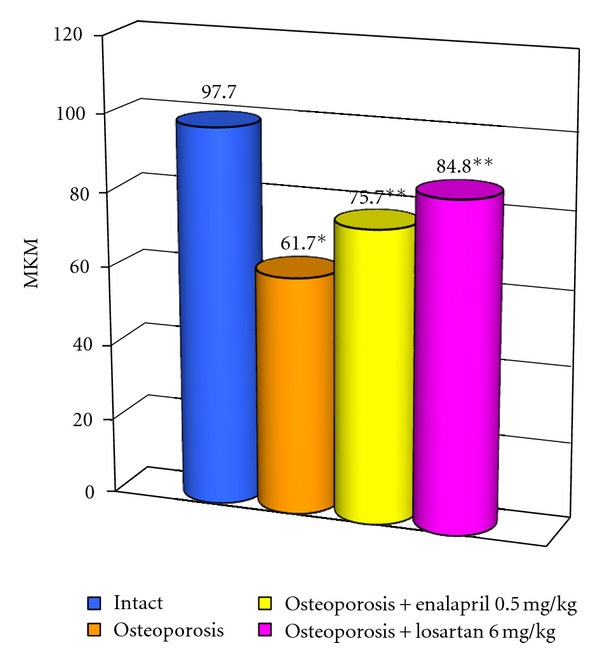
Effect of enalapril and losartan on the mean width of bone trabeculae in the proximal metaphysis of the femur after eight weeks of bilateral ovariectomy. *P < 0.05 compared with a group of intact animals; **P < 0.05 compared with a group of rats with experimental osteoporosis MKM = *μ*m.

Microscopy and histomorphometry sections of the femur in rats receiving treatment found no microfractures of trabeculae, the preservation of bone structure, and a large width of bone trabeculae other than in rats with osteoporosis who were not receiving treatment (Figure 3).

Studying the individual indicators of microcirculation and the average width of bone trabeculae in the proximal metaphysis of the femur in all groups of animals, there was attention to the presence of a certain relationship between these two parameters. To confirm the presence of such dependence, Pearson's correlation coefficient (*r*) was calculated between the level of the microcirculation and an average width of bone trabeculae. In addition, for comparison of paired values in the group, a dot diagram of the width of bone trabeculae on the level of regional microcirculation was built and charts of the regression line were made. The following values were obtained: in the group of intact animals *r* = 0.7, in group of rats with osteoporosis *r* = 0.6, in females which were administered enalapril *r* = 0.4, and in the group with losartan *r* = 0.8. These coefficient values indicate a fairly close direct correlation between the level of microcirculation and an average width of bone trabeculae in the proximal metaphysic of femur in rats of all groups.

The results of LDF in rats with experimental fractures showed that the consolidation of fractures in the background of generalized osteoporosis (*n* = 20) has average level of the microcirculation in the callus (69.7 ± 5.9 PU) which is significantly lower than that in rats without osteoporosis (*n* = 20) (87.6 ± 6.3 PU). In animals treated with enalapril, the average level of microcirculation was recorded exceeding over than that of the animals with femur fractures without osteoporosis (102.2 ± 5.4 PU). However, the average value of the microcirculation in fracture zone in rats treated with losartan was equal to 123.1 ± 5.0 PU, which is also significantly higher than those in rats with femoral fractures without osteoporosis and higher than in rats treated with enalapril.

**Figure 4:**
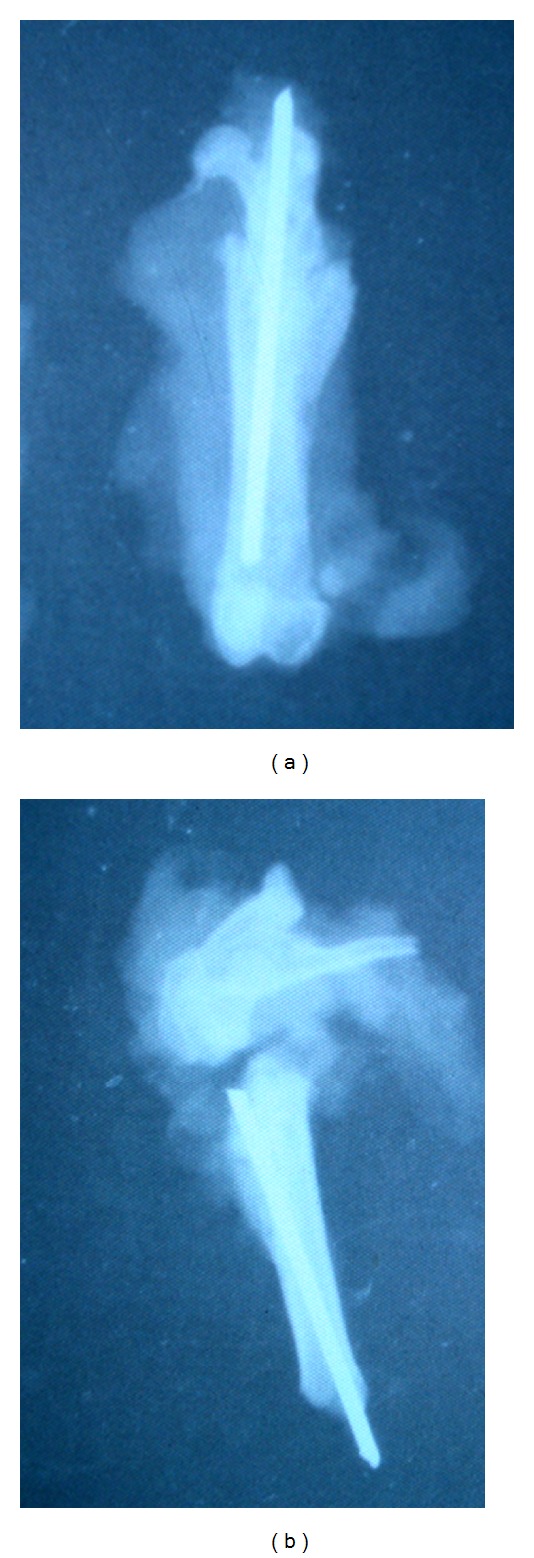
Examples of X-ray with consolidation and nonunion of fractures. (a) Consolidation fracture of the femur 4 weeks after osteotomy. (b) Nonunion fracture.

In the visual evaluation of the fracture zone of proximal metaphysis of femur, the majority of rats in all groups after four weeks after the fracture showed signs of callus formation but also observed poor outcome, nonunion fracture (Figure 4).

In studying the results of the consolidation of fractures, four weeks after osteotomy, it was found that in intact animals nonunion fractures were in four cases and in rats with osteoporosis eight cases out of 20. In animals that were administered the study drug, there was only one nonunion fracture in rats receiving enalapril 0.5 mg/kg. In the group with losartan 6 mg/kg, consolidation of all fractures was observed. Thus, the consolidation of experimental osteoporotic fractures of the proximal metaphysis of femur in female Wistar rats after twelve weeks after ovariectomy occurs in the face of declining circulation in the fracture zone, which will undoubtedly have a negative impact on the results of union of fractures, leading to a significant increase in the number of unsatisfactory outcomes. Therapy with enalapril and losartan in a period of twelve weeks after ovariectomy shows increased rates of microcirculation in the zone between fragments in consolidation of fractures of the proximal metaphysis of the femur in rats. Thus, the studied drugs significantly increased the frequency of consolidation of experimental fractures.

## 5. Conclusion

1. Eight weeks after ovariectomy in female Wistar rats, endothelial dysfunction of vessels of microcirculation in bone tissue develops, with a significant (40%) deterioration in regional blood flow, which in turn leads to a thinning of bone trabeculae (average 39.4%) and the emergence of microfractures in them, therefore causing osteoporosis.
2. Enalapril dose 0.5 mg/kg and losartan dose 6 mg/kg effectively prevent the decrease in regional microcirculation of the bone, which allows to support the processes of bone remodeling in osteoporosis. Losartan has a more pronounced osteoprotective effect.
3. Comparison of consolidation of experimental osteoporotic fractures of the proximal metaphysis of femoral with similar fractures in rats without osteoporosis shows in the former a decrease level of microcirculation in bone (20%), which negatively affects the outcome of union of fractures leading to doubling the number of poor outcomes of consolidation.
4. Enalapril and losartan increase the rate of microcirculation in the fracture zone of proximal metaphysis of the femur to an exceeding value in rats with experimental osteoporotic fractures by 30% and 40%, respectively, as well as increase the number of positive results of consolidation of fractures. Moreover, the effect of losartan on the microcirculation in fracture zone exceeds a similar effect of enalapril.

## Figures and Tables

**Table 1:** Index of Microcirculation, coefficient of endothelial dysfunction and the average width of bone trabecular in the proximal metaphysis of the femur, the once received treatment with the studied medication is compared with control group.

Animal group	Level of microcirculation (in PE)	Coefficient of endothelial dysfunction	The average width of bone trabeculae (in *μ*m)
The intact with fracture	100.5 ± 4.4	1.3 ± 0.2	97.68 ± 1.0
Osteoporosis + fracture	61.5 ± 3.7	2.4 ± 0.2	61.68 ± 1.2
Osteoporosis + fracture + enalapril 0.5 mg/kg	93.3 ± 4.4	1.6 ± 0.1	75.65 ± 0.7
Osteoporosis + fracture + losartan 6 mg/kg	100.0 ± 2.3	1.5 ± 0.2	84.76 ± 0.6

**Table 2:** The average parameters of microcirculation, the width of the bone trabecule in the bony callus and rate of consolidation of the experimental osteoporotic fractures of the proximal part of rats femur, the once received treatment with the studied medication is compared with control group.

Animal group	Level of microcirculation (in PE)	The average width of bone trabeculae (in *μ*m)	Number of consolidated fractures
The intact with fracture	87.64 ± 6.3	92.93 ± 1.6	16 out of 20
Osteoporosis + fracture	69.7 ± 5.9	59.02 ± 1.7	12 out of 20
Osteoporosis + fracture + enalapril 0.5 mg/kg	102.23 ± 5.4	76.98 ± 1.4	19 out of 20
Osteoporosis fracture + losartan 6 mg/kg	123.11 ± 4.97	81.88 ± 1.8	20 out of 20
